# The efficacy and safety of different doses of glucocorticoid for autoimmune hepatitis: A systematic review and meta-analysis

**DOI:** 10.1097/MD.0000000000018313

**Published:** 2019-12-27

**Authors:** Chi Zhang, Shan-Shan Wu, Xiao-Qin Dong, Zhao Wu, Hong Zhao, Gui-Qiang Wang

**Affiliations:** a Department of Infectious Disease, Center for Liver Disease, Peking University First Hospital, Xicheng District; b National Clinical Research Center of Digestive Diseases, Beijing Friendship Hospital, Capital Medical University; c Peking University International Hospital, Beijing; d The Collaborative Innovation Center for Diagnosis and Treatment of Infectious Diseases, Zhejiang University, Hangzhou, Zhejiang, China.

**Keywords:** autoimmune hepatitis, biochemical remission rate, endpoint events incidence, glucocorticoid, side effects

## Abstract

**Background:**

Glucocorticoid as the standard treatment of autoimmune hepatitis has been recommended with different doses. The purpose of this study is to compare the efficacy and safety of high and low doses for clinical practice.

**Methods:**

Medline, Embase, and Cochrane Library were searched until January 16th, 2019 for cohort studies or randomized controlled trials in patients with autoimmune hepatitis. Glucocorticoid 60 mg/d or 1 mg/kg/d was defined as high dose and 40 to 50 mg/d or 0.5 mg/d as low dose. Outcome of interests includes the incidence of the biochemical remission, adverse event, and endpoint events. Double arcsine method with a random-effect model was used to combine the incidence. Potential heterogeneity was explored by meta-regression and subgroup analysis.

**Results:**

Overall, 25 studies (3305 patients) were included, with 10 studies in the high dose group and 15 in low dose group. The biochemical remission rate in the high and low dose group was 0.79 (95% confidence interval [CI] [0.72, 0.85]) and 0.72 (95% CI [0.65, 0.78]), respectively. The incidence of endpoint events and adverse event in the high were slightly higher (0.03, 95% CI [0.02, 0.04]; 0.42, 95% CI [0.30, 0.53]) than that of the low dose group (0.01, 95% CI [0.00, 0.01]; 0.39, 95% CI [0.15, 0.63]).

**Conclusions:**

For autoimmune hepatitis patients, 60 mg/d or 1 mg/kg/d of glucocorticoid gives higher biochemical remission rate and higher incidence of endpoint events and adverse events.

## Abstract 2

Supplemental Digital Content is available in the text

## 1 Introduction

Autoimmune hepatitis (AIH) is an inflammatory liver condition characterized by abnormal autoimmune reactions that can occur in individuals of all ages, sexes, and races. The diagnosis for AIH is based on histological abnormalities, characteristic clinical and biochemical findings, serum auto-antibodies, and abnormal levels of serum globulins. AIH was the first liver disease for which an effective therapeutic intervention, corticosteroid treatment, was convincingly demonstrated in controlled clinical trials. While corticosteroids alone or in combination with azathioprine are effective and prolong survival.^[1]^

However, different guidelines recommend different doses of glucocorticoid. The American Association for the Study of Liver Diseases (AASLD) guideline^[2]^ and European Association for the Study of the Liver (EASL) guideline^[1]^ recommend Prednisone alone (60 mg daily) or a lower dose of prednisone (30 mg daily) in conjunction with azathioprine (50 mg daily). The Chinese guideline recommend the initial dose of prednisone (prednisolone) at 30 to 40 mg/d combined with azathioprine.^[3]^

Different doses are recommended in these guidelines, but it is not known whether there is a difference in efficacy. The greater the dose of glucocorticoid, the higher the side effects.[^4^,^5^] The EASL guideline reported that glucocorticoid therapy had numerous adverse events, and severe adverse events occur mainly at doses >20 mg/d for more than 18 months and lead to treatment discontinuation in about 15% of patients.^[1]^ Wang et al study^[6]^ which involved 82 patients with decompensated cirrhosis indicated the incidence of liver transplantation or death in patients receiving low dose glucocorticoid therapy (10 mg/d–50 mg/d) was significantly lower than those receiving no glucocorticoid therapy (14.1% vs 50.0%, *P* < .05). Yasui et al study^[7]^ of 28 patients with liver failure or fulminant hepatitis caused by AIH, 25 patients received 40 to 60 mg prednisolone or 1000 mg prednisolone pulse therapy, and 3 patients without glucocorticoid therapy (mortality rate was 100%) and 25 people with glucocorticoid therapy (mortality rate was 28%). Because AIH is not a common disease, as its prevalence ranges from 16 to 18 cases per 100,000 people in Europe,[^8^,^9^,^10^,^11^,^12^,^13^] most of the current studies are small sample studies. Especially in patients with cirrhosis and liver failure caused by AIH, it is difficult to evaluate the safety and efficacy of high dose and low dose of glucocorticoid with small sample size. Therefore, we need to do this meta-analysis to clarify the safety and efficacy of high dose and low dose glucocorticoid.

## 2 Methods

This meta-analysis was based on previous published studies which have declared ethical approvals and did not add new data, so ethical approval was not conducted.

This systematic review and meta-analysis was reported in accordance with the preferred reporting items for systematic reviews and meta-analyses (PRISMA) statement^[14]^ (Supplemental Digital Content [Appendix 8] [Table], which illustrates the PRISMA statement) and was registered at International Prospective Register of Systematic Reviews (PROSPERO number CRD42019121951).

### 2.1 Search strategy and selection criteria

Medline, Embase, and the Cochrane Central Register of Controlled Trials were searched from inception to January 16th, 2019 using the following keywords

Autoimmune hepatitis, therapeutics, prednisone, prednisolone, glucocorticoid, azathioprine (Supplemental Digital Content [Appendix 1] [Table], which demonstrates the full details about the search strategy). Two reviewers independently screened the potential publication titles and abstracts, and reviewed the full-text of the eligible articles. AIH was diagnosed according to the “Diagnostic Scoring System of the International Autoimmune Hepatitis Group” or modified AIH International Study Group criteria.^[15]^

The studies included in this meta-analysis should meet the following criteria:

(1) The type of study was either cohort study or randomized controlled study.
(2) Participants were patients with definite diagnosis of AIH.
(3) Treatment with glucocorticoid or glucocorticoid combined with azathioprine.

Exclusion criteria were as follows:

(1) Patients with overlap syndrome,
(2) Patients with recurrent AIH after liver transplantation.
(3) Reviews, editorials, letters, guidelines, and protocols were excluded.
(4) Language was limited in English.

In addition, if 2 or more studies were published based on the same sample, the article with the highest quality was included. Any studies which did not meet the above criteria were excluded.

In Chinese guideline of AIH, 40 to 50 mg/d or 0.5 mg/d is often recommended, but in EASL and AASLD guideline recommend 60 mg/d. According to previous studies, for AIH, the greater the dose of glucocorticoid, the greater the side effects, but whether the higher the dose of glucocorticoid, the better the control of liver disease is unknown. Because of the low incidence of AIH, according to the literature we searched, there is little literature on acute AIH, liver failure or liver cirrhosis. Therefore, we divide the proportion of this kind of special population into different groups, to get the preliminary results about this kind of special population, and also provide a reference for the follow-up research. So, we define the “acute onset ≥50% subgroup” as: the number of acute patients in the study accounted for more than 50% of the total number of patients included in the study. Similarly, we define the “cirrhosis onset ≥30% subgroup” as: the number of cirrhosis patients in the study accounted for more than 30% of the total number of patients included in the study. And the “liver failure and fulminant hepatitis onset ≥15% subgroup” as: the number of liver failure or fulminant hepatitis patients in the study accounted for more than 15% of the total number of patients included in the study.

### 2.2 Data extraction

Two authors (CZ and SSW) independently extracted the information using a standardized form for each study, including author's name, year of publication, region, study type, sample size, onset condition (acute or chronic onset, cirrhosis or not, liver failure/fulminant hepatitis or not), sex, mean age of participants, initial dose of glucocorticoid (prednisone or prednisolone), initial dose of azathioprine, observation time, number of adverse events of glucocorticoid, number of patients with biochemical remission, number of patients with end-point events (liver transplantation or death). Any discrepancies were resolved by discussion with senior investigators (HZ, GQW).

### 2.3 Quality assessment

As all included studies were longitudinal study with only AIH patients who received either lower dose or higher dose treatment, the methodological quality of the studies was assessed using an 11-item checklist which was recommended by Agency for Healthcare Research and Quality (AHRQ).^[16]^ An item would be scored “0” if it was answered “NO” or “UNCLEAR;” if it was answered “YES,” then the item scored “1.” Article quality was assessed as follows: low quality = 0–3; moderate quality = 4–7; high quality = 8–11.

### 2.4 Outcome measure

The primary outcome of interest was biochemical remission which mean normalization of hepatic enzymes, mainly aspartate aminotransferase and alanine aminotransferase (ALT). The secondary outcome of interest included endpoint events which mean liver transplantation or death, and adverse events of glucocorticoid.

### 2.5 Statistical analysis

The incidence of each outcome and 95% confidence interval (CI) was calculated as effect measurement. Considering the low incidence of endpoint events, the double arcsine transformation was used to calculate the incidence of endpoint events.^[17]^ Heterogeneity was expected, so all analyses were performed with a random-effects model.^[18]^ *Q*-statistics and Cochrane *Q* test were used to assess heterogeneity between studies, where *P* < .10 was regarded to be statistically significant.[^18^,^19^] The *I*-square was calculated to describe the percent of observed variation across studies caused by heterogeneity. Subgroup analysis and meta-regression analysis were performed to explore potential sources of heterogeneity. Factors examined including glucocorticoid dosage (high dose vs low dose), study type (randomized controlled trial (RCT) vs non-RCT), age (children vs adult), region (Europe and America vs non-Europe and America), observation time (≤1 year vs 1 year–5 years vs >5 years), onset acute proportion (<50% vs ≥50%), onset cirrhosis proportion (<30% vs ≥30%), onset liver failure or fulminant hepatitis proportion (<15% vs ≥15%). In addition, to examine the impact of a single study on total effect, sensitivity analysis by leaving out 1 study each time was carried out.

Funnel plot and Begg test were used to examine the potential publication bias. *P* ≤ .05 was considered to be statistically significant. All analyses were conducted using STATA 12.0.

## 3 Results

### 3.1 Search results and study characteristics

**Figure 1:**
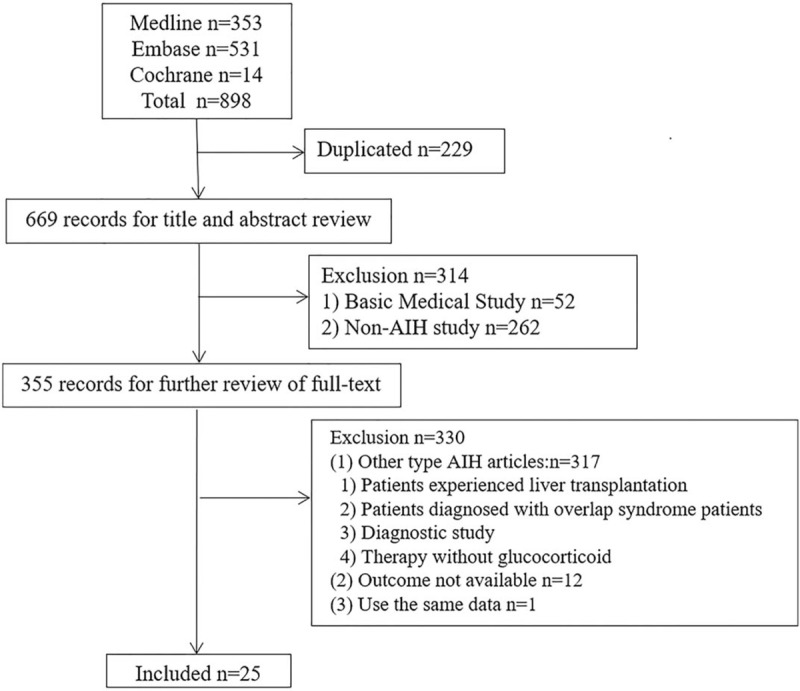
Flowchart for study selection in the meta-analysis.

There were 898 studies identified from Medline, Embase, and Cochrane Library. Among these studies, we removed 229 duplicate studies. Three hundred fourteen studies were excluded by reviewing title and abstract, due to basic medical study or non-AIH study, and 330 studies were excluded by reviewing full-text, due to other type AIH articles or outcome not available or use the same data. So, 25 studies (including 3305 patients) met the inclusion criteria (Supplemental Digital Content [Appendix 2] [reference], which is the full reference list) and all of them met the diagnostic criteria for AIH mentioned above. Among the 25 studies published from 2001 to 2018, there were 3 randomized controlled studies, 10 high dose studies, 13 in Europe and the United States, 18 in adults. Because age is expressed differently in different original studies, therefore, we converted them into mean age by statistical method.[^20^,^21^,^22^] The mean age of the included studies ranged from 8.8 to 58.0 years (children group mean age 8.8–14.2 years, adult group mean age 37.2–58.0 years). Observation time ranged from 0.06 to 16.0 years. The flowchart shows the detailed process of selection (Fig. 1) and the detailed information is presented in Table 1.

**Table 1:**
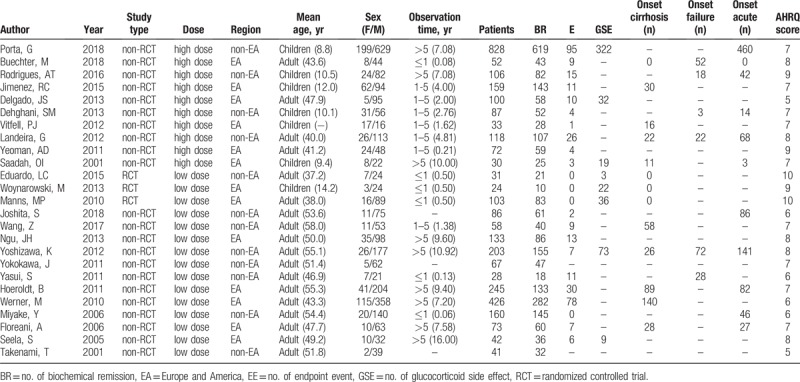
Characteristics of studies included in the meta-analysis.

### 3.2 Methodological quality assessment

All of the selected studies were assessed for methodological quality by AHRQ. The AHRQ score of each study was presented in Table 1 and Supplemental Digital Content (Appendix 3) (Table), which illustrates the detailed AHRQ score. Ten studies[^23^,^24^,^25^,^26^,^27^,^28^,^29^,^30^,^31^,^32^] were of high quality and 15 studies[^6^,^7^,^33^,^34^,^35^,^36^,^37^,^38^,^39^,^40^,^41^,^42^,^43^,^44^,^45^] were of moderate quality. There were no studies with low quality.

### 3.3 Incidence of biochemical remission, endpoint events, and adverse events

**Figure 2:**
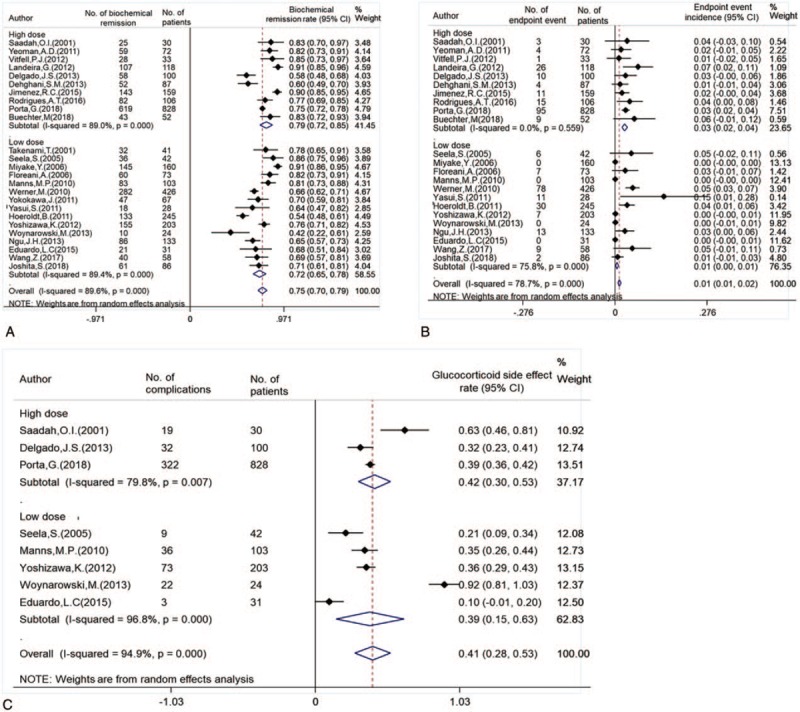
The biochemical remission rate (A) and endpoint event incidence (B) and adverse events incidence (C) of different doses of glucocorticoid were used in AIH patients.

As shown in Figure 2A, the combined biochemical remission rate was 0.75 (95% CI 0.70, 0.79), in the high and low dose group was 0.79 (95% CI 0.72, 0.85) and 0.72 (95% CI 0.65, 0.78), respectively. The combined endpoint events (liver transplantation and death) rate (Fig. 2B) was 0.01 (95% CI 0.01, 0.02), in the high and low dose group was 0.03 (95% CI 0.02, 0.04) and 0.01 (95% CI 0.00, 0.01), respectively. The combined adverse events (Fig. 2C) incidence of glucocorticoid was 0.41 (95% CI 0.28, 0.53), in the high dose group was higher than that of low dose group (0.42 [95% CI 0.30, 0.53] vs 0.39 [95% CI 0.15, 0.63]). Although there are different side effects, such as weight gain, full-moon face, heavy pigmentation, peptic ulcer, and other symptoms like Cushing syndrome, these are tolerable to patients without obvious discomfort. But there are also serious complications, such as cryptococcal meningitis and aseptic necrosis of hip joint in 1 patient each in Seela et al^[32]^ study and intracerebral hemorrhage and femoral head necrosis in Yoshizawa et al^[28]^ study.

### 3.4 Subgroup analysis and heterogeneity analysis and meta-regression

#### 3.4.1 Biochemical remission rate

**Figure 3:**
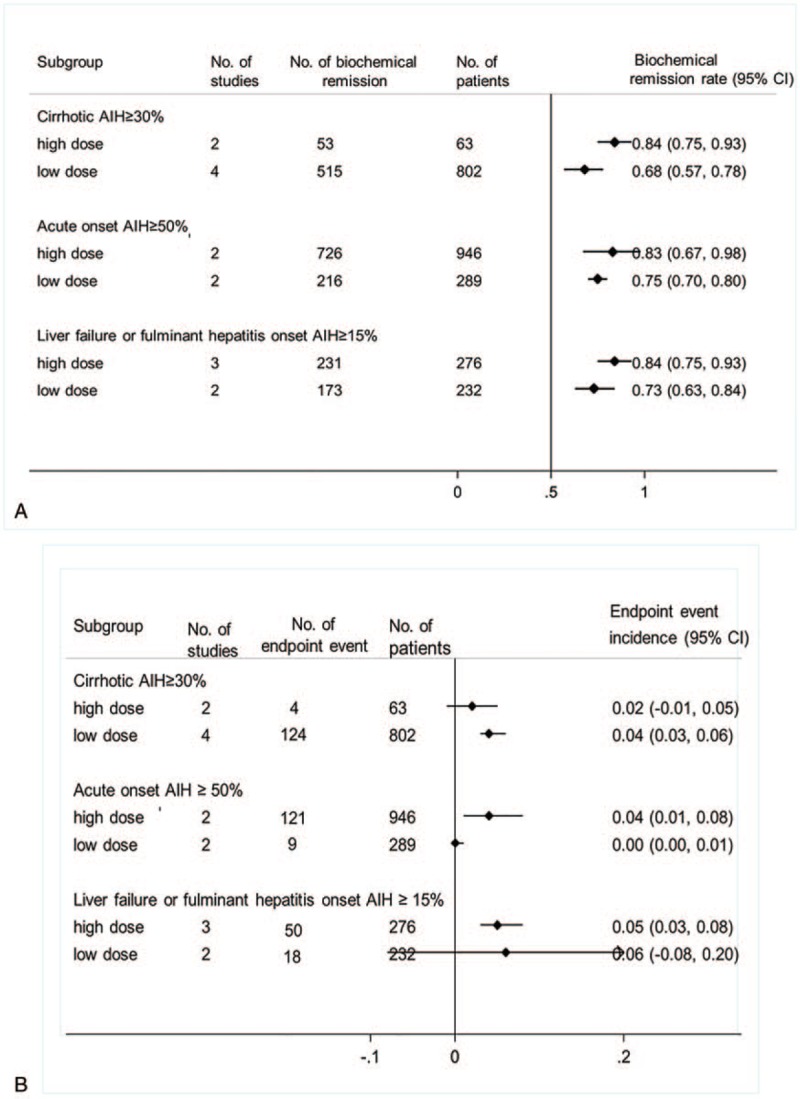
The biochemical remission rate (A) and endpoint event incidence (B) of different doses of glucocorticoid were used in cirrhotic AIH patients, acute onset AIH patients and liver failure or fulminant hepatitis onset AIH. AIH = autoimmune hepatitis.

As shown in Table 2 and Supplemental Digital Content (Appendix 4) (Figure), which illustrates the forest plot of biochemical remission rate, the biochemical remission rate was higher in non-RCT group than the RCT group. There was no significant different among different age groups and regional groups. The biochemical remission rate of observation more than 5 years was slightly lower than that of 1 to 5 years and less than 1 year. As shown in Figure 3A, in the acute onset ≥50% subgroup, cirrhosis onset ≥30% subgroup, and the liver failure or fulminant hepatitis onset ≥15% subgroup the high dose was higher than low dose. The children and adults have different clinical characteristics in AIH. Whether there is a difference in their response to glucocorticoid is unknown. Therefore, it is necessary to separate children from adults. However, in this analysis, the biochemical remission rates of children and adults are 0.75 (95% CI [0.66, 0.84]) and 0.74 (95% CI [0.68, 0.80]), respectively. There is no statistical difference in the results. Detailed forest plots are shown in Supplemental Digital Content (Appendix 4.4) (Figure) which demonstrates biochemical remission rate classified by age subgroup.

**Table 2:**
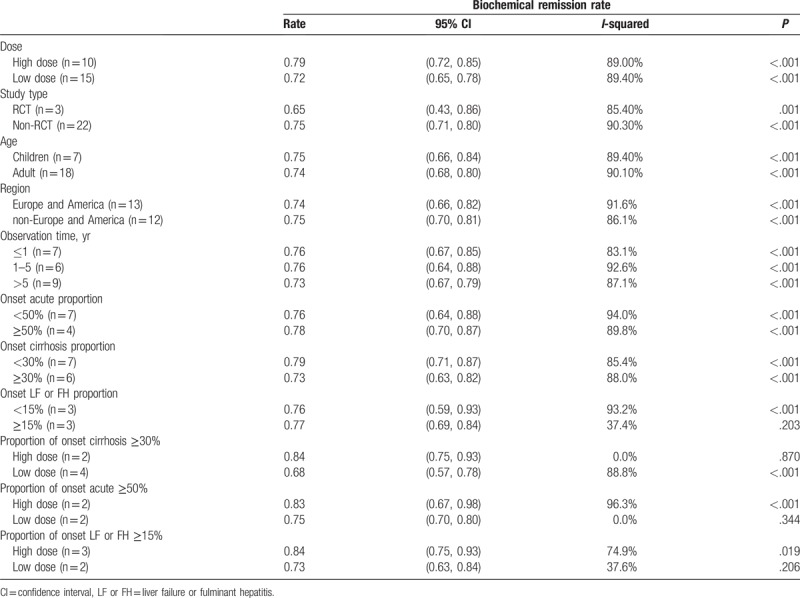
Summary of the biochemical remission rate of high and low doses of glucocorticoid were used in different subgroup AIH patients.

For heterogeneity analysis of biochemical remission rates, subgroup analysis (Table 2, Supplemental Digital Content [Appendix 4] [Figure] which demonstrate forest plot of biochemical remission rate) and meta-regression (Table 4) were used. Regrettably, subgroup analysis did not find the main source of heterogeneity, and influence analysis (Supplemental Digital Content [Appendix 7.3] [Figure] which was the metaninf command plot of biochemical remission rate) was also used to examine the impact of excluding a study on the overall merger effect, and the main source of heterogeneity was not found. The univariate meta-regression method was used (Table 4), publication year, study type, dose, region, proportion of onset cirrhosis, proportion of onset failure, proportion of onset acute, and observation time were also analyzed, all of the *P* > .05. Finally, only the random effect model was used to reduce the heterogeneity.

#### 3.4.2 Endpoint event incidence

As shown in Table 3 and Supplemental Digital Content (Appendix 5) (Figure) which demonstrate forest plot of endpoint event incidence, the endpoint event incidence was higher in high dose group than the low dose group. There was no significant different among different age groups, regional groups, onset acute proportion group, and onset liver failure or fulminant hepatitis proportion group. The endpoint event incidence of observation less than 1-year group was slightly lower than that of 1 to 5 years and more than 5 years group. As shown in Figure 3B, in the acute onset ≥50% subgroup the high dose was higher than low dose. In the cirrhosis onset ≥30% subgroup, the high dose was lower than low dose and in the liver failure or fulminant hepatitis onset ≥15% subgroup, the high dose and low dose had no significant different. Like the biochemical remission rate, the endpoint event incidence in children and adults were 0.02 (95% CI [0.00, 0.03]) and 0.01 (95% CI [0.00, 0.01]), respectively, and with no statistical differences. Detailed forest plots are shown in Supplemental Digital Content (Appendix 5.4) (Figure) which illustrated endpoint event incidence classified by age subgroup.

**Table 3:**
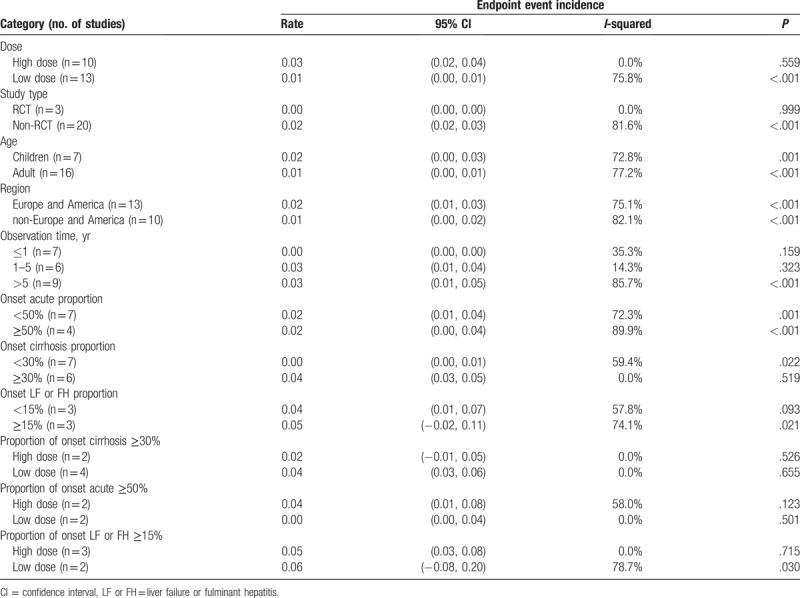
Summary of the endpoint event incidence of high and low doses of glucocorticoid were used in different subgroup AIH patients.

**Table 4:**
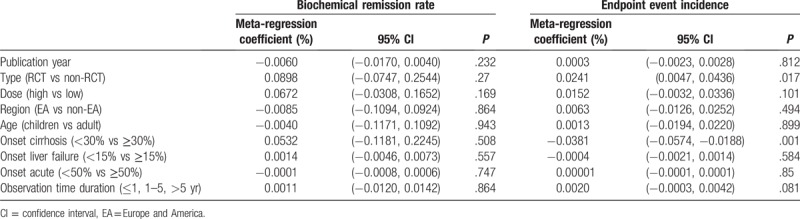
Meta-regression results of biochemical remission rate and endpoint events incidence.

Subgroup analysis (Table 3), influence analysis (Supplemental Digital Content [Appendix 7.7] [Figure] which illustrated the metaninf command plot of endpoint event incidence) and meta-regression (Table 4) were also used to analyze the heterogeneity of endpoint events incidence. The results of subgroup analysis (Table 3) showed that the heterogeneity could be reduced, in the large dose group (*P* = .559, *I* = 0.0%), RCT group (*P* = .999, *I* = 0.0%), observation time and the proportion of cirrhosis patients also could be reduced differently. In univariate meta-regression analyses (Table 4), study type (*P* = .017) and proportion of onset cirrhosis (*P* = .001) were the main source of heterogeneity. Publication year, dose, region, proportion of onset failure, proportion of onset acute, and observation time were also analyzed, all of the *P* > .05. The results of influence analysis (Supplemental Digital Content [Appendix 7.7] [Figure]) show that excluding Miyake et al study,^[42]^ study research, the combined effect of endpoint events has some changes).

### 3.5 Publication bias

For biochemical remission rate, endpoint event and adverse events of glucocorticoid, funnel plot (Supplemental Digital Content [Appendix 6] [Figure] which was the funnel plot of this meta-analysis) showed a little bit asymmetry. However, Begg test (Supplemental Digital Content [Appendix 7] [Figure] which demonstrated publication bias test and sensitivity analysis) did not detect any publication bias with all of the *P*-values greater than .05.

## 4 Discussion

Many guidelines[^1^,^2^,^3^,^46^,^47^] recommend glucocorticoid as the standard treatment for AIH, but there is no evidence-based data on the efficacy and safety of different doses of glucocorticoid. In this systematic review and meta-analysis of 25 studies and 3305 patients, we assessed incidence of biochemical remission, endpoint events, and adverse events of different doses of glucocorticoid. Our meta-analysis showed that high doses of glucocorticoid had high biochemical remission rates, high incidence of adverse events, and endpoint events.

The results of this study suggest that high doses of glucocorticoid may be beneficial in patients with acute hepatitis or liver failure caused by AIH. Previous guidelines[^1^,^3^] have shown that when patients with AIH are in seriously condition, such as elevated ALT, elevated bilirubin or liver fibrosis, and so on, it often indicates that the liver is in a state of over-activation of immune system, and the immune system attacks its own hepatocytes. To inhibit the over-activated immune system, a slightly higher dose of glucocorticoid can better control the abnormal immune activation state, and provide time for the control of the disease and the regeneration and repair of liver cells.

Anand et al study^[48]^ of AIH causing acute on chronic liver failure showed that the 90-day survival rate in the glucocorticoid group (40 mg daily) was significantly higher than that in the glucocorticoid-free group (75.0% vs 48.1%), and the length of hospitalization in intensive care unit was also shorter. Both this study and Anand et al study^[48]^ have shown that glucocorticoid was beneficial in patients with liver failure or fulminant hepatitis. In recent Buechter et al study,^[23]^ all patients were treated with 1 mg/kg/d of glucocorticoid. The 28-day mortality or liver transplantation rate was 17.3%. No recurrence of liver transplantation or death occurred during subsequent follow-up. Compared with the above 2 studies, high doses of glucocorticoid seem to have a higher survival rate in patients with AIH-induced liver failure. In the subgroup with acute onset of more than 50%, the biochemical remission rate and the incidence of end-point events in the high dose group were higher than those in the low dose group. Traditionally, the higher the biochemical remission rate, the lower the incidence of end-point events, but this meta-analysis yielded the opposite results. The reason may be that high doses of glucocorticoid can increase the incidence of infection or other adverse reactions of glucocorticoid, which is one of the reasons why the incidence of end-point events in the high dose group is higher than that in the low dose group. To control acute or severe illness, high dose of glucocorticoid is beneficial to biochemical remission rate. However, due to the difference between patient population and follow-up events, rigorous prospective research results need to be designed.

The results of this study suggest that high doses of glucocorticoid may be beneficial in patients with cirrhosis caused by AIH. In the subgroup with cirrhosis onset of more than 30%, the biochemical remission rate in the high dose group was higher than that in the low dose group, and the incidence of end-point events in the high dose group was lower than that in the low dose group. Not surprisingly, different studies have different doses of glucocorticoid for AIH with onset of liver cirrhosis, such as the Chinese guidelines^[3]^ recommend the choice of glucocorticoid monotherapy. The initial dose of prednisone (prednisolone) was properly reduced (20–30 mg/d). Wang et al study^[6]^ showed that patients with decompensated cirrhosis returned to compensatory cirrhosis in a higher proportion of patients treated with glucocorticoid than those in the nonglucocorticoid group. But 6 of the 9 deaths or liver transplants were caused by infection. Whether these infections are associated with glucocorticoid use has not been raised in the study.

Some limitations have to be noted. First, the language included in the study is limited to English, which might be leading to publication bias. However, Begg test did not indicate any publication bias. Second, although subgroup analysis and meta-regression were carried out as far as possible, there was still some heterogeneity on outcomes, it may be that the incidence of AIH is not high, so most of the studies included are non-RCT. Finally, all studies included were longitudinal studies with only 1 group treated with high dose or low dose glucocorticoid. All of them did not have a comparable control group, which might reduce the evidence quality. Thus, our results should be interpreted with caution. Further randomized control trials with enough sample size are needed to validate our results.

## 5 Conclusion

In the treatment of AIH, 60 mg/d or 1 mg/kg/d glucocorticoid has a high biochemical remission rate, but at the same time, it is accompanied by obvious side effects and endpoint events. For patients with acute AIH, liver failure or cirrhosis, 60 mg/d or 1 mg/kg/d of glucocorticoid may be considered.

## Author contributions

**Conceptualization:** Chi Zhang, Shan Shan Wu, Hong Zhao, Gui-Qiang Wang.

**Data curation:** Chi Zhang, Shan Shan Wu, Xiao-Qing Dong, Zhao Wu.

**Formal analysis:** Shan Shan Wu.

**Funding acquisition:** Hong Zhao, Gui-Qiang Wang.

**Investigation:** Shan Shan Wu, Xiao-Qing Dong, Zhao Wu, Hong Zhao.

**Methodology:** Shan Shan Wu, Hong Zhao.

**Project administration:** Gui-Qiang Wang.

**Resources:** Hong Zhao, Gui-Qiang Wang.

**Software:** Chi Zhang, Shan Shan Wu, Xiao-Qing Dong, Zhao Wu.

**Supervision:** Hong Zhao, Gui-Qiang Wang.

**Validation:** Hong Zhao, Gui-Qiang Wang.

**Visualization:** Xiao-Qing Dong, Hong Zhao, Gui-Qiang Wang.

**Writing – original draft:** Chi Zhang, Shan Shan Wu.

**Writing – review and editing:** Shan Shan Wu, Xiao-Qing Dong, Zhao Wu, Hong Zhao.

## Supplementary Material

Supplemental Digital Content

## Supplementary Material

Supplemental Digital Content

## Supplementary Material

Supplemental Digital Content

## Supplementary Material

Supplemental Digital Content

## Supplementary Material

Supplemental Digital Content

## Supplementary Material

Supplemental Digital Content

## Supplementary Material

Supplemental Digital Content

## Supplementary Material

Supplemental Digital Content
