# Supplementary material for: The efficacy and safety of different doses of glucocorticoid for autoimmune hepatitis: A systematic review and meta-analysis
Source: Medicine (Baltimore). 2019 Dec 27;98(52):e18313. doi: 10.1097/MD.0000000000018313 (PMC6946338; doi:10.1097/MD.0000000000018313)
Supplement: Supplemental Digital Content [file medi-98-e18313-s002.docx]

**The efficacy and safety of different dose glucocorticoid for autoimmune hepatitis: a systematic review and meta-analysis**

Chi Zhang, Shan-Shan Wu, Xiao-Qin Dong, Zhao Wu,

Hong Zhao, Gui-Qiang Wang

**Supplementary appendix to the manuscript**

**Contents of supplementary appendix**

| Appendix 1 | Search strategy and results |
| --- | --- |
| Appendix 2 | References for included studies |
| Appendix 3 | Quality assessment of included studies by Agency for Healthcare Research and Quality (AHRQ) |
| Appendix 4 | The forest plot of biochemical remission rate classified by subgroup included in the meta-analysis |
| Appendix 5 | The forest plot of endpoint event incidence classified by subgroup included in the meta-analysis |
| Appendix 6 | The funnel plot of this meta-analysis |
| Appendix 7 | Publication bias test and sensitivity analysis |
| Appendix 8 | PRISMA 2009 Checklist |

**Appendix 1**

**Search strategy and results**

**Number of citations by each database and trial register searched**

| **Databases and trial registers** | **Citations** |
| --- | --- |
| **Databases** |  |
| Medline | 353 |
| Embase | 531 |
| Cochrane Library | 14 |
| **Total databases** | **898** |

**Search strategy for Pubmed-Medline**

#1 “Hepatitis, Autoimmune”[Mesh]

#2 “Therapeutics”[Mesh]

#3 ”Prednisone”[Mesh]

#4 ”Prednisolone”[Mesh]

#5 ”Glucocorticoids”[Mesh]

#6 ”Azathioprine”[Mesh]

#7 #2 OR #3 OR #4 OR #5 OR #6

#8 #1 AND #7

#9 (#1 AND #7) Sort by: Best Match Filters: Case Reports; Congress;Government Document; Guideline; Interview; Meta-Analysis; News; Systematic Reviews; Comment; Review

#10 #8 NOT #9

#11 #10 Sort by: Best Match Filters: Full text; Humans; English

**Search strategy for Embase**

#1. 'autoimmune hepatitis'/exp

#2. 'therapy'/exp

#3. 'prednisone'/exp

#4. 'prednisolone'/exp

#5. 'glucocorticoid'/exp

#6. 'azathioprine'/exp

#7. #2 OR #3 OR #4 OR #5 OR #6

#8. #1 AND #7

#9. #8 AND [humans]/lim AND [english]/lim

#10. #8 AND [humans]/lim AND [english]/lim AND ([conference abstract]/lim OR [conference paper]/lim OR [conference review]/lim OR [editorial]/lim OR [erratum]/lim OR [letter]/lim OR [note]/lim OR [review]/lim OR [short survey]/lim)

#11. #9 NOT #10

#12. #9 NOT #10

#13. #11 AND ('hepatitis b'/de OR 'hepatitis c'/de OR 'primary biliary cirrhosis'/de OR 'primary sclerosing cholangitis'/de)

#14. #12 NOT #13

#15. #14 AND ('controlled clinical trial'/de OR 'controlled study'/de OR 'observational study'/de OR 'prospective study'/de OR 'randomized controlled trial'/de OR 'retrospective study'/de)

**Search strategy for Cochrane Library**

#1 MeSH descriptor: [Hepatitis, Autoimmune] explode all trees

#2 MeSH descriptor: [Therapeutics] explode all trees

#3 MeSH descriptor: [Prednisone] explode all trees

#4 MeSH descriptor: [Prednisolone] explode all trees

#5 MeSH descriptor: [Glucocorticoids] explode all trees

#6 MeSH descriptor: [Azathioprine] explode all trees

#7 #2 OR #3 OR #4 OR #5 OR #6

#8 #1 AND #7
