# Supplementary material for: The efficacy and safety of different doses of glucocorticoid for autoimmune hepatitis: A systematic review and meta-analysis
Source: Medicine (Baltimore). 2019 Dec 27;98(52):e18313. doi: 10.1097/MD.0000000000018313 (PMC6946338; doi:10.1097/MD.0000000000018313)
Supplement: Supplemental Digital Content [file medi-98-e18313-s003.docx]

**Appendix 2**

**References for included studies**

(1-25)

1. Porta G, Carvalho ED, Santos JL, Gama J, Borges CV, Seixas RBPM, et al. Autoimmune hepatitis in 828 Brazilian children and adolescents: clinical and laboratory findings, histological profile, treatments, and outcomes. Jornal de pediatria. 2018.

2. Joshita S, Yoshizawa K, Umemura T, Ohira H, Takahashi A, Harada K, et al. Clinical features of autoimmune hepatitis with acute presentation: a Japanese nationwide survey. Journal of gastroenterology. 2018;53(9):1079-88.

3. Buechter M, Manka P, Heinemann FM, Lindemann M, Baba HA, Schlattjan M, et al. Potential triggering factors of acute liver failure as a frst manifestation of autoimmune hepatitis-a single center experience of 52 adult patients. World journal of gastroenterology. 2018;24(13):1410-8.

4. Wang Z, Sheng L, Yang Y, Yang F, Xiao X, Hua J, et al. The Management of Autoimmune Hepatitis Patients with Decompensated Cirrhosis: Real-World Experience and a Comprehensive Review. Clinical Reviews in Allergy and Immunology. 2017;52(3):424-35.

5. Rodrigues AT, Liu PM, Fagundes ED, Queiroz TC, de Souza Haueisen Barbosa P, Silva SL, et al. Clinical Characteristics and Prognosis in Children and Adolescents With Autoimmune Hepatitis and Overlap Syndrome. Journal of pediatric gastroenterology and nutrition. 2016;63(1):76-81.

6. Nct. Possible Role of Chloroquine to Induce a Complete Remission in the Treatment of Autoimmune Hepatitis: a Randomized Trial. [Https://clinicaltrialsgov/show/nct02463331](https://clinicaltrialsgov/show/nct02463331). 2015.

7. Jiménez-Rivera C, Ling SC, Ahmed N, Yap J, Aglipay M, Barrowman N, et al. Incidence and characteristics of autoimmune hepatitis. Pediatrics. 2015;136(5):e1237-e48.

8. Woynarowski M, Nemeth A, Baruch Y, Koletzko S, Melter M, Rodeck B, et al. Budesonide versus prednisone with azathioprine for the treatment of autoimmune hepatitis in children and adolescents. The Journal of pediatrics. 2013;163(5):1347-53.e1.

9. Ngu JH, Gearry RB, Frampton CM, Stedman CA. Predictors of poor outcome in patients w ith autoimmune hepatitis: a population-based study. Hepatology (Baltimore, Md). 2013;57(6):2399-406.

10. Delgado JS, Vodonos A, Malnick S, Kriger O, Wilkof-Segev R, Delgado B, et al. Autoimmune hepatitis in southern Israel: a 15-year multicenter study. Journal of digestive diseases. 2013;14(11):611-8.

11. Dehghani SM, Haghighat M, Imanieh MH, Honar N, Negarestani AM, Malekpour A, et al. Autoimmune hepatitis in children: Experiences in a Tertiary Center. Iranian Journal of Pediatrics. 2013;23(3):302-8.

12. Yoshizawa K, Matsumoto A, Ichijo T, Umemura T, Joshita S, Komatsu M, et al. Long-term outcome of Japanese patients with type 1 autoimmune hepatitis. Hepatology (Baltimore, Md). 2012;56(2):668-76.

13. Vitfell-Pedersen J, Jørgensen MH, Müller K, Heilmann C. Autoimmune hepatitis in children in Eastern Denmark. Journal of pediatric gastroenterology and nutrition. 2012;55(4):376-9.

14. Landeira G, Morise S, Fassio E, Ramonet M, Álvarez E, Caglio P, et al. Effect of cirrhosis at baseline on the outcome of type 1 autoimmune hepatitis. Annals of hepatology. 2012;11(1):100-6.

15. Yokokawa J, Kanno Y, Saito H, Abe K, Takahashi A, Yokokawa H, et al. Risk factors associated with relapse of type 1 autoimmune hepatitis in Japan. Hepatology Research. 2011;41(7):641-6.

16. Yeoman AD, Westbrook RH, Zen Y, Maninchedda P, Portmann BC, Devlin J, et al. Early predictors of corticosteroid treatment failure in icteric presentations of autoimmune hepatitis. Hepatology (Baltimore, Md). 2011;53(3):926-34.

17. Yasui S, Fujiwara K, Yonemitsu Y, Oda S, Nakano M, Yokosuka O. Clinicopathological features of severe and fulminant forms of autoimmune hepatitis. Journal of gastroenterology. 2011;46(3):378-90.

18. Hoeroldt B, McFarlane E, Dube A, Basumani P, Karajeh M, Campbell MJ, et al. Long-term outcomes of patients with autoimmune hepatitis managed at a nontransplant center. Gastroenterology. 2011;140(7):1980-9.

19. Werner M, Wallerstedt S, Lindgren S, Almer S, Bjornsson E, Bergquist A, et al. Characteristics and long-term outcome of patients with autoimmune hepatitis related to the initial treatment response. Scandinavian journal of gastroenterology. 2010;45(4):457-67.

20. Manns MP, Woynarowski M, Kreisel W, Lurie Y, Rust C, Zuckerman E, et al. Budesonide induces remission more effectively than prednisone in a controlled trial of patients with autoimmune hepatitis. Gastroenterology. 2010;139(4):1198-206.

21. Miyake Y, Iwasaki Y, Sakaguchi K, Shiratori Y. Clinical features of Japanese male patients with type 1 autoimmune hepatitis. Alimentary Pharmacology and Therapeutics. 2006;24(3):519-23.

22. Floreani A, Niro G, Rosa Rizzotto E, Antoniazzi S, Ferrara F, Carderi I, et al. Type I autoimmune hepatitis: Clinical course and outcome in an Italian multicentre study. Alimentary Pharmacology and Therapeutics. 2006;24(7):1051-7.

23. Seela S, Sheela H, Boyer JL. Autoimmune hepatitis type 1: safety and efficacy of prolonged medical therapy. Liver international : official journal of the International Association for the Study of the Liver. 2005;25(4):734-9.

24. Takenami T, Sakaguchi K, Nishimura M, Miyake Y, Miyashita M, Terao M, et al. Therapeutic effects of azathioprine in combination with low-dose prednisolone in patients with intractable autoimmune hepatitis type 1. Acta medica Okayama. 2001;55(6):341-7.

25. Saadah OI, Smith AL, Hardikar W. Long-term outcome of autoimmune hepatitis in children. Journal of Gastroenterology and Hepatology (Australia). 2001;16(11):1297-302.
