# Supplementary material for: The efficacy and safety of different doses of glucocorticoid for autoimmune hepatitis: A systematic review and meta-analysis
Source: Medicine (Baltimore). 2019 Dec 27;98(52):e18313. doi: 10.1097/MD.0000000000018313 (PMC6946338; doi:10.1097/MD.0000000000018313)
Supplement: Supplemental Digital Content [file medi-98-e18313-s004.docx]

**Appendix 3**

**Quality assessment of included studies by Agency for Healthcare Research and Quality (AHRQ)**

| Item | Porta,G.  2018 | Buechter,M  2018 | Rodrigues,A.T. 2016 | Jimenez,R.C.  2015 | Delgado,J.S. 2013 |
| --- | --- | --- | --- | --- | --- |
| 1) Define the source of information (survey, record review) | Yes | Yes | Yes | Yes | Yes |
| 2) List inclusion and exclusion criteria for exposed and unexposed subjects (cases and controls) or refer to previous publications | No | Yes | Yes | Yes | No |
| 3) Indicate time period used for identifying patients | Yes | Yes | Yes | Yes | Yes |
| 4) Indicate whether or not subjects were consecutive if not population-based | Unclear | Unclear | Unclear | No | Unclear |
| 5) Indicate if evaluators of subjective components of study were masked to other aspects of the status of the participants | No | No | Unclear | Unclear | Unclear |
| 6) Describe any assessments undertaken for quality assurance purposes (e.g., test/retest of primary outcome measurements) | Yes | Yes | Yes | Yes | No |
| 7) Explain any patient exclusions from analysis | Yes | Yes | Yes | Unclear | Unclear |
| 8) Describe how confounding was assessed and/or controlled. | Unclear | Unclear | Yes | Unclear | Unclear |
| 9) If applicable, explain how missing data were handled in the analysis | Yes | Yes | Yes | Yes | Yes |
| 10) Summarize patient response rates and completeness of data collection | Yes | Yes | Yes | Yes | Yes |
| 11) Clarify what follow-up, if any, was expected and the percentage of patients for which incomplete data or follow-up was obtained | Yes | Yes | Yes | Unclear | Yes |
| Score | 7 | 8 | 9 | 7 | 5 |

| Item | Dehghani,S.M. 2013 | Vitfell,P.J.  2012 | Landeira,G.  2012 | Yeoman,A.D.  2011 | Saadah,O.I. 2001 |
| --- | --- | --- | --- | --- | --- |
| 1) Define the source of information (survey, record review) | Yes | Yes | Yes | Yes | Yes |
| 2) List inclusion and exclusion criteria for exposed and unexposed subjects (cases and controls) or refer to previous publications | No | No | Yes | Unclear | No |
| 3) Indicate time period used for identifying patients | Unclear | Yes | Unclear | Yes | Yes |
| 4) Indicate whether or not subjects were consecutive if not population-based | Unclear | Unclear | Unclear | Yes | Unclear |
| 5) Indicate if evaluators of subjective components of study were masked to other aspects of the status of the participants | Yes | Yes | No | Unclear | No |
| 6) Describe any assessments undertaken for quality assurance purposes (e.g., test/retest of primary outcome measurements) | Yes | Yes | Yes | Yes | Yes |
| 7) Explain any patient exclusions from analysis | Yes | Unclear | Yes | Yes | Yes |
| 8) Describe how confounding was assessed and/or controlled. | Unclear | Unclear | Yes | Yes | Yes |
| 9) If applicable, explain how missing data were handled in the analysis | Yes | Yes | Yes | Yes | Unclear |
| 10) Summarize patient response rates and completeness of data collection | Yes | Yes | Yes | Yes | Yes |
| 11) Clarify what follow-up, if any, was expected and the percentage of patients for which incomplete data or follow-up was obtained | Yes | Yes | Yes | Yes | Yes |
| Score | 7 | 7 | 8 | 9 | 7 |

| Item | Eduardo,L.C 2015 | Woynarowski,M. 2013 | Manns,M.P.  2010 | Joshita,S.  2018 | Wang,Z.  2017 |
| --- | --- | --- | --- | --- | --- |
| 1) Define the source of information (survey, record review) | Yes | Yes | Yes | Yes | Unclear |
| 2) List inclusion and exclusion criteria for exposed and unexposed subjects (cases and controls) or refer to previous publications | Yes | Unclear | Yes | Unclear | Yes |
| 3) Indicate time period used for identifying patients | Yes | Yes | Yes | Yes | Yes |
| 4) Indicate whether or not subjects were consecutive if not population-based | Unclear | Yes | Yes | No | Yes |
| 5) Indicate if evaluators of subjective components of study were masked to other aspects of the status of the participants | Yes | Yes | No | Yes | Yes |
| 6) Describe any assessments undertaken for quality assurance purposes (e.g., test/retest of primary outcome measurements) | Yes | Unclear | Yes | Unclear | Unclear |
| 7) Explain any patient exclusions from analysis | Yes | Yes | Yes | Yes | Unclear |
| 8) Describe how confounding was assessed and/or controlled. | Yes | Yes | Yes | Yes | Yes |
| 9) If applicable, explain how missing data were handled in the analysis | Yes | Yes | Yes | Unclear | Unclear |
| 10) Summarize patient response rates and completeness of data collection | Yes | Yes | Yes | Yes | Yes |
| 11) Clarify what follow-up, if any, was expected and the percentage of patients for which incomplete data or follow-up was obtained | Yes | Yes | Yes | Unclear | Yes |
| Score | 10 | 9 | 10 | 6 | 7 |

| Item | Ngu,J.H. 2013 | Yoshizawa,K. 2012 | Yokokawa,J.  2011 | Yasui,S.  2011 | Hoeroldt,B. 2011 |
| --- | --- | --- | --- | --- | --- |
| 1) Define the source of information (survey, record review) | Yes | Yes | Yes | No | Unclear |
| 2) List inclusion and exclusion criteria for exposed and unexposed subjects (cases and controls) or refer to previous publications | Unclear | Yes | No | Unclear | Yes |
| 3) Indicate time period used for identifying patients | Yes | Yes | Yes | Yes | Yes |
| 4) Indicate whether or not subjects were consecutive if not population-based | Yes | Unclear | Yes | Yes | Yes |
| 5) Indicate if evaluators of subjective components of study were masked to other aspects of the status of the participants | Yes | Yes | Yes | Yes | Unclear |
| 6) Describe any assessments undertaken for quality assurance purposes (e.g., test/retest of primary outcome measurements) | Yes | Yes | Yes | Yes | Unclear |
| 7) Explain any patient exclusions from analysis | No | Unclear | Unclear | Unclear | Yes |
| 8) Describe how confounding was assessed and/or controlled. | Yes | Yes | Yes | Yes | Yes |
| 9) If applicable, explain how missing data were handled in the analysis | Unclear | Unclear | Unclear | Unclear | Yes |
| 10) Summarize patient response rates and completeness of data collection | Yes | Yes | Yes | Yes | Yes |
| 11) Clarify what follow-up, if any, was expected and the percentage of patients for which incomplete data or follow-up was obtained | Yes | Yes | Unclear | Unclear | No |
| Score | 8 | 8 | 7 | 6 | 7 |

| Item | Werner,M. 2010 | Miyake,Y.  2006 | Floreani,A.  2006 | Seela,S.  2005 | Takenami,T. 2001 |
| --- | --- | --- | --- | --- | --- |
| 1) Define the source of information (survey, record review) | Yes | Yes | Yes | Yes | No |
| 2) List inclusion and exclusion criteria for exposed and unexposed subjects (cases and controls) or refer to previous publications | No | No | Unclear | Unclear | No |
| 3) Indicate time period used for identifying patients | Unclear | Unclear | Yes | Yes | Yes |
| 4) Indicate whether or not subjects were consecutive if not population-based | Yes | Yes | Yes | Yes | Yes |
| 5) Indicate if evaluators of subjective components of study were masked to other aspects of the status of the participants | Yes | Yes | Yes | Yes | Yes |
| 6) Describe any assessments undertaken for quality assurance purposes (e.g., test/retest of primary outcome measurements) | Yes | Yes | Yes | Yes | Unclear |
| 7) Explain any patient exclusions from analysis | Unclear | Unclear | Unclear | Unclear | Unclear |
| 8) Describe how confounding was assessed and/or controlled. | Yes | Yes | Yes | Yes | Yes |
| 9) If applicable, explain how missing data were handled in the analysis | No | Unclear | Unclear | Unclear | No |
| 10) Summarize patient response rates and completeness of data collection | Yes | Yes | Yes | Yes | Yes |
| 11) Clarify what follow-up, if any, was expected and the percentage of patients for which incomplete data or follow-up was obtained | No | No | No | Yes | No |
| Score | 6 | 6 | 7 | 8 | 5 |
