# Supplementary material for: The efficacy and safety of different doses of glucocorticoid for autoimmune hepatitis: A systematic review and meta-analysis
Source: Medicine (Baltimore). 2019 Dec 27;98(52):e18313. doi: 10.1097/MD.0000000000018313 (PMC6946338; doi:10.1097/MD.0000000000018313)
Supplement: Supplemental Digital Content [file medi-98-e18313-s005.docx]

**Appendix 4**

**The forest plot of biochemical remission rate classified by subgroup included in the meta-analysis**

4.1 biochemical remission rate

4.2 biochemical remission rate classified by dose subgroup

4.3 biochemical remission rate classified by study type subgroup

4.4 biochemical remission rate classified by age subgroup

4.5 biochemical remission rate classified by region subgroup

4.6 biochemical remission rate classified by observation time subgroup

4.7 biochemical remission rate classified by onset acute proportion subgroup

4.8 biochemical remission rate classified by onset cirrhosis proportion subgroup

4.9 biochemical remission rate classified by onset LF or FH proportion subgroup

4.10 biochemical remission rate classified by dose subgroup(proportion of onset cirrhosis≥30%)

4.11 biochemical remission rate classified by dose subgroup(proportion of onset acute≥50%)

4.12 biochemical remission rate classified by dose subgroup(proportion of onset LF or FH≥15%)

4.1 biochemical remission rate

4.2 biochemical remission rate classified by dose subgroup

4.3 biochemical remission rate classified by study type subgroup

4.4 biochemical remission rate classified by age subgroup

4.5 biochemical remission rate classified by region subgroup

4.6 biochemical remission rate classified by observation time subgroup

4.7 biochemical remission rate classified by onset acute proportion subgroup

4.8 biochemical remission rate classified by onset cirrhosis proportion subgroup

4.9 biochemical remission rate classified by onset LF or FH proportion subgroup

4.10 biochemical remission rate classified by dose subgroup(proportion of onset cirrhosis≥30%)

4.11 biochemical remission rate classified by dose subgroup(proportion of onset acute≥50%)

4.12 biochemical remission rate classified by dose subgroup(proportion of onset LF or FH≥15%)
