# Supplementary material for: The efficacy and safety of different doses of glucocorticoid for autoimmune hepatitis: A systematic review and meta-analysis
Source: Medicine (Baltimore). 2019 Dec 27;98(52):e18313. doi: 10.1097/MD.0000000000018313 (PMC6946338; doi:10.1097/MD.0000000000018313)
Supplement: Supplemental Digital Content [file medi-98-e18313-s006.docx]

**Appendix 7**

**Publication bias test and sensitivity analysis**

7.1 the Begg plot of biochemical remission rate

7.2 the Egger plot of biochemical remission rate

7.3 the metaninf command plot of biochemical remission rate

7.4 the Galbraith plot of biochemical remission rate

7.5 the Begg plot of endpoint event incidence

7.6 the Egger plot of endpoint event incidence

7.7 the metaninf command plot of endpoint event incidence

7.8 the Galbraith plot of endpoint event incidence

7.9 the Begg plot of glucocorticoid side effect

7.10 the Egger plot of glucocorticoid side effect

7.11 the metaninf command plot of glucocorticoid side effect

7.12 the Galbraith plot of glucocorticoid side effect

7.1 the Begg plot of biochemical remission rate

7.2 the Egger plot of biochemical remission rate

7.3 the metaninf command plot of biochemical remission rate

7.4 the Galbraith plot of biochemical remission rate

7.5 the Begg plot of endpoint event incidence

7.6 the Egger plot of endpoint event incidence

7.7 the metaninf command plot of endpoint event incidence

7.8 the Galbraith plot of endpoint event incidence

7.9 the Begg plot of glucocorticoid side effect

7.10 the Egger plot of glucocorticoid side effect

7.11 the metaninf command plot of glucocorticoid side effect

7.12 the Galbraith plot of glucocorticoid side effect
