# Supplementary material for: The efficacy and safety of different doses of glucocorticoid for autoimmune hepatitis: A systematic review and meta-analysis
Source: Medicine (Baltimore). 2019 Dec 27;98(52):e18313. doi: 10.1097/MD.0000000000018313 (PMC6946338; doi:10.1097/MD.0000000000018313)
Supplement: Supplemental Digital Content [file medi-98-e18313-s007.docx]

**Appendix 5**

**The forest plot of endpoint event incidence classified by subgroup included in the meta-analysis**

5.1 endpoint event incidence

5.2 endpoint event incidence classified by dose subgroup

5.3 endpoint event incidence classified by study type subgroup

5.4 endpoint event incidence classified by age subgroup

5.5 endpoint event incidence classified by region subgroup

5.6 endpoint event incidence classified by observation time subgroup

5.7 endpoint event incidence classified by onset acute proportion subgroup

5.8 endpoint event incidence classified by onset cirrhosis proportion subgroup

5.9 endpoint event incidence classified by onset LF or FH proportion subgroup

5.10 endpoint event incidence classified by dose subgroup(proportion of onset cirrhosis≥30%)

5.11 endpoint event incidence classified by dose subgroup(proportion of onset acute≥50%)

5.12 endpoint event incidence classified by dose subgroup(proportion of onset LF or FH≥15%)

5.1 endpoint event incidence

5.2 endpoint event incidence classified by dose subgroup

5.3 endpoint event incidence classified by study type subgroup

5.4 endpoint event incidence classified by age subgroup

5.5 endpoint event incidence classified by region subgroup

5.6 endpoint event incidence classified by observation time subgroup

5.7 endpoint event incidence classified by onset acute proportion subgroup

5.8 endpoint event incidence classified by onset cirrhosis proportion subgroup

5.9 endpoint event incidence classified by onset LF or FH proportion subgroup

5.10 endpoint event incidence classified by dose subgroup(proportion of onset cirrhosis≥30%)

5.11 endpoint event incidence classified by dose subgroup(proportion of onset acute≥50%)

5.12 endpoint event incidence classified by dose subgroup(proportion of onset LF or FH≥15%)
