# Supplementary material for: The efficacy and safety of different doses of glucocorticoid for autoimmune hepatitis: A systematic review and meta-analysis
Source: Medicine (Baltimore). 2019 Dec 27;98(52):e18313. doi: 10.1097/MD.0000000000018313 (PMC6946338; doi:10.1097/MD.0000000000018313)
Supplement: Supplemental Digital Content [file medi-98-e18313-s008.docx]

**Appendix 6**

**The funnel plot of this meta-analysis**

6.1 The funnel plot of biochemical remission rate

6.2 The funnel plot of endpoint event incidence

6.3 The funnel plot of glucocorticoid side effect

6.1 The funnel plot of biochemical remission rate

6.2 The funnel plot of endpoint event incidence

6.3 The funnel plot of glucocorticoid side effect
